# The Effectiveness of Smartphone App–Based Interventions for Assisting Smoking Cessation: Systematic Review and Meta-analysis

**DOI:** 10.2196/43242

**Published:** 2023-04-20

**Authors:** Yi-Qiang Guo, Yuling Chen, Annette DeVito Dabbs, Ying Wu

**Affiliations:** 1 School of Nursing Capital Medical University Beijing China; 2 Johns Hopkins University School of Nursing Baltimore, MD United States; 3 School of Nursing University of Pittsburgh Pittsburgh, PA United States

**Keywords:** smartphone app, smoking cessation, meta-analysis, eHealth, mHealth, smoking, application, intervention, effectiveness, electronic, adult, pharmacotherapy

## Abstract

**Background:**

Smoking is a leading cause of premature death globally. Quitting smoking reduces the risk of all-cause mortality by 11%-34%. Smartphone app–based smoking cessation (SASC) interventions have been developed and are widely used. However, the evidence for the effectiveness of smartphone-based interventions for smoking cessation is currently equivocal.

**Objective:**

The purpose of this study was to synthesize the evidence for the effectiveness of smartphone app–based interventions for smoking cessation.

**Methods:**

We conducted a systematic review and meta-analysis of the effectiveness of smartphone interventions for smoking cessation based on the Cochrane methodology. An electronic literature search was performed using the Cochrane Library, Web of Science, PubMed, Embase, PsycINFO, China National Knowledge Infrastructure, and Wanfang databases to identify published papers in English or Chinese (there was no time limit regarding the publication date). The outcome was the smoking abstinence rate, which was either a 7-day point prevalence abstinence rate or a continuous abstinence rate.

**Results:**

A total of 9 randomized controlled trials involving 12,967 adults were selected for the final analysis. The selected studies from 6 countries (the United States, Spain, France, Switzerland, Canada, and Japan) were included in the meta-analysis between 2018 and 2022. Pooled effect sizes (across all follow-up time points) revealed no difference between the smartphone app group and the comparators (standard care, SMS text messaging intervention, web-based intervention, smoking cessation counseling, or apps as placebos without real function; odds ratio [OR] 1.25, 95% CI 0.99-1.56, *P*=.06, *I*^2^=73.6%). Based on the subanalyses, 6 trials comparing smartphone app interventions to comparator interventions reported no significant differences in effectiveness (OR 1.03, 95% CI 0.85-1.26, *P*=.74, *I*^2^=57.1%). However, the 3 trials that evaluated the combination of smartphone interventions combined with pharmacotherapy compared to pharmacotherapy alone found higher smoking abstinence rates in the combined intervention (OR 1.79, 95% CI 1.38-2.33, *P*=.74, *I*^2^*=*7.4%). All SASC interventions with higher levels of adherence were significantly more effective (OR 1.48, 95% CI 1.20-1.84, *P*<.001, *I*^2^*=*24.5%).

**Conclusions:**

This systematic review and meta-analysis did not support the effectiveness of delivering smartphone-based interventions alone to achieve higher smoking abstinence rates. However, the efficacy of smartphone-based interventions increased when combined with pharmacotherapy-based smoking cessation approaches.

**Trial Registration:**

PROSPERO CRD42021267615; https://www.crd.york.ac.uk/prospero/display_record.php?RecordID=267615

## Introduction

Smoking is a leading cause of premature death globally. Among tobacco-related deaths, cigarette smoking accounts for half of the people who regularly smoke [1-3]. Quitting smoking reduces the risk of all-cause mortality by 11%-34% [4]. However, an estimated 1.3 billion individuals worldwide continue to use tobacco products [5]. Many interventions for promoting smoking cessation have been implemented worldwide [6-11]. Behavioral change therapies and pharmacotherapeutics are the most recommended approaches to assist smoking cessation, both individually and in combination, and are effective in increasing smoking cessation in adults [12].

Health information technology–based interventions (such as a computer, tablet, or smartphone) [13] are associated with increased smoking cessation in the general population [9,13]. The number of smartphone users is increasing [14], and smartphone app–based interventions have been developed and are widely used. The advantages of using a smartphone app–based intervention include the ease of use, as they can be used anywhere at any time, the ability to provide visual information via video, and the ability to deliver interventions on a large scale. Moreover, they also have the ability to tailor messages in terms using specifically provided information (such as age, mood, time, and nicotine independence scores) by using algorithms. However, the evidence for the effectiveness of smartphone-based interventions for smoking cessation is currently controversial. To evaluate the cumulative effects of smartphone app–based smoking cessation (SASC) interventions on smoking cessation, researchers conducted a Cochrane systematic review in 2019. However, the level of evidence was insufficient because the review only included 5 randomized controlled trials (RCTs) with small sample sizes, which might have affected the pooled effect of the meta-analysis [9].

Recently, more RCTs of SASC interventions with larger sample sizes have been conducted [6,15-18], and have provided opportunities to explore the effectiveness of SASC interventions with greater confidence. However, the results of those RCTs were conflicting. Five studies found that the SASC interventions did not lead to a higher smoking abstinence rate [19-23]; in contrast, other studies reported that the SASC interventions increased smoking abstinence [18,24-26]. Hence, our study aimed to explore the pooled effects of SASC interventions in promoting smoking abstinence.

## Methods

This study was conducted according to the Preferred Reporting Items for Systematic Review and Meta-Analyses (ie, PRISMA) guidelines [27]. The protocol of this study was registered in the International Prospective Register of Systematic Reviews (CRD42021267615).

### Search Strategy

We performed a comprehensive literature search to identify RCTs related to the SASC intervention for smoking cessation. Databases, including the Cochrane Library, PubMed, Embase, PsycINFO, and Web of Science, were searched for published papers in English. For Chinese publications, we searched the China National Knowledge Infrastructure and Wanfang databases, which are the primary databases in China. In addition, we manually searched through the reference lists of study reports to identify additional eligible studies and contacted the authors as needed for more information.

The search terms included a combination of keywords related to mobile health and smoking cessation. The search strategies used in PubMed, Embase, PsycINFO, Web of Science, and the Cochrane Library are listed in the Multimedia Appendix 1. The key terms included *telemedicine*, *mobile health*, *mHealth*, *eHealth*, *telehealth*, *digital health*, *mobile phone*, *mobile device*, *cellphone*, *smartphone*, smartphone app*, cellphone app*, mobile app*, *smartphone-based*, *cellphone-based*, *mobile phone-based*, portable electronic app*, portable software app* or smoking, *smoking abstinence*, *smoking cessation*, *tobacco abstinence*, *tobacco use cessation*, *tobacco quitting*, quit* smok*, give up smok*, and stop* smok*. In addition, search filters were used to extract only RCTs. The searches were conducted independently by 2 investigators and they used EndNote (version X9; Clarivate Analytics) to import and manage selected articles.

### Study Selection Criteria

#### Overview

Studies were selected if they evaluated the effect of SASC interventions, were guided by health behavior change theory, and used an RCT design, including publications, dissertations, and conference papers based on the Population–Intervention–Comparison–Outcome (ie, PICO) method. Although we did not specify the exclusion of pilot studies in the research proposal, according to the National Institutes of Health, the goal of pilot work is not to test hypotheses about the effects of an intervention, but rather to assess the feasibility and acceptability of an approach to be used in a larger-scale study [28]. Thus, we calculated the sample size based on the smoking abstinence rate [24]. As a result, each group of 113 provides 80% power to detect a 15% reduction in smoking abstinence rate with 2-sided testing and set at .05 and the following equivalence tests for the difference of 2 proportions in a cluster-randomized design. For this review, pilot studies (each group with fewer than 113 participants) were excluded.

#### PICO Elements

##### Population

Eligible participants included (1) anyone who smokes ≥5 cigarettes per day and (2) active users of a smartphone. Exclusion criteria included studies with participants younger than 18 years.

##### Intervention

Smartphone app–assisted interventions to support smoking cessation based on health behavior change theory. According to Gitlin [29], without a theory, behavioral interventions will have limited success. The theory addresses 3 broad essential questions: why the intervention should work (the development phase), how the intervention works (the evaluation phase), and how the intervention works in real settings (the implementation phase). Most of the studies reported that the intervention was designed based on the behavioral change theory. For the reports that did not cite a guiding theory, the corresponding authors were contacted to confirm the use of the health behavior change theory prior to selecting the study for inclusion in the review.

##### Comparison

The comparison group could be any other type of supportive smoking cessation, including standard care, SMS text messaging intervention, web-based intervention, or smoking cessation counseling.

##### Outcomes

The outcome was the smoking abstinence rate, reported as either a 7-day point prevalence abstinence rate or a continuous abstinence rate, assessed at least 3 months after baseline using objective or self-report measures. According to the Nicotine and Tobacco Subcommittee on Biochemical Verification [30], biochemical validation is not always necessary in smoking cessation studies since levels of misrepresentation are generally low (0%-8.8%), thus reducing the likelihood of bias in reporting. All studies measuring smoking abstinence, including solely self-reported outcomes, were included.

### Data Extraction

Titles and abstracts were retrieved using the search strategy by an author (YQG), and the duplicates were removed. Based on the selection criteria, 2 reviewers (YQG and YW) independently screened the titles and abstracts. The authors (YQG and YLC) reviewed the full texts, and any disagreements were resolved through discussion until consensus was reached or by consulting the third author (YW). The full texts of the selected papers were reviewed, and the reasons for exclusion were documented.

Two reviewers (YQG and YLC) then independently extracted the data from each eligible study. The extracted information included: (1) study characteristics, including authors’ names, year of publication, country setting, study design, sample characteristics, name of the smartphone app–assisted interventions to support smoking cessation, comparator, details of the intervention, type of outcome measurements, retention rate, funding, and conflict of interest, follow-up period, whether or not the RCT protocol was published or registered; and (2) the outcomes of interest included biochemically verified smoking abstinence rate or self-reported smoking abstinence rates from different smoking cessation interventions after 3, 6, and 12 months.

### Risk-of-Bias Assessment

Two reviewers (YQG and YLC) independently assessed the quality of studies according to the Cochrane Collaboration risk of the bias assessment tool, RoB 2 [31,32], which includes 5 domains corresponding to specific types of bias: (1) bias arising from the randomization process, (2) bias due to deviations from intended interventions, (3) bias due to missing outcome data, (4) bias in the measurement of the outcome, and (5) bias in the selection of the reported result. A judgment of “low risk,” “high risk,” or “some concern” of bias was assigned to each domain. Disagreements between the 2 reviewers were resolved by consulting with the third reviewer (YW). A final decision was made after the 3 reviewers reached a consensus.

### Data Synthesis and Analysis

We analyzed the pooled effects of continuous or point-prevalence tobacco abstinence rates as the primary outcomes. Subgroup analyses were performed based on different follow-up points, interventions (solely SASC interventions or SASC combined with pharmacotherapies), levels of adherence, and outcome measure methods.

The meta-analysis was conducted using RevMan (version 5.4; Cochrane Training), a desktop version of Review Manager software. The funnel plot, a simple scatter plot of the intervention effect estimates from individual studies against some measure of each study’s size or precision, and the Begg and Egger tests were conducted with Stata (version 17 SE; StataCorp) software to determine the presence of publication bias. The number of people randomized to the intervention and control groups was extracted to calculate the odds ratio (OR). The pooled effect sizes are presented in forest plots.

Heterogeneity among studies was assessed using the chi-square test, and *I*^2^ values were used to determine heterogeneity across studies, attributing to the proportion of total variation, in which an *I*^2^>50% indicated substantial heterogeneity of effects, and random-effects models were applied. If the *I*^2^ was <50%, fixed-effects models were selected [33]. A leave-one-out sensitivity analysis was done by removing each trial from the analysis to explore whether an outlier trial had an undue influence on the meta-analysis. Subgroup analyses were performed based on different follow-up time points. Additionally, we separated the RCTs by whether the SASC interventions were used alone or with adjuvants of clinical smoking cessation therapy. All statistical tests were 2-tailed, and a *P* value of less than .05 was considered statistically significant.

## Results

### Results of the Literature Search

**Figure 1:**
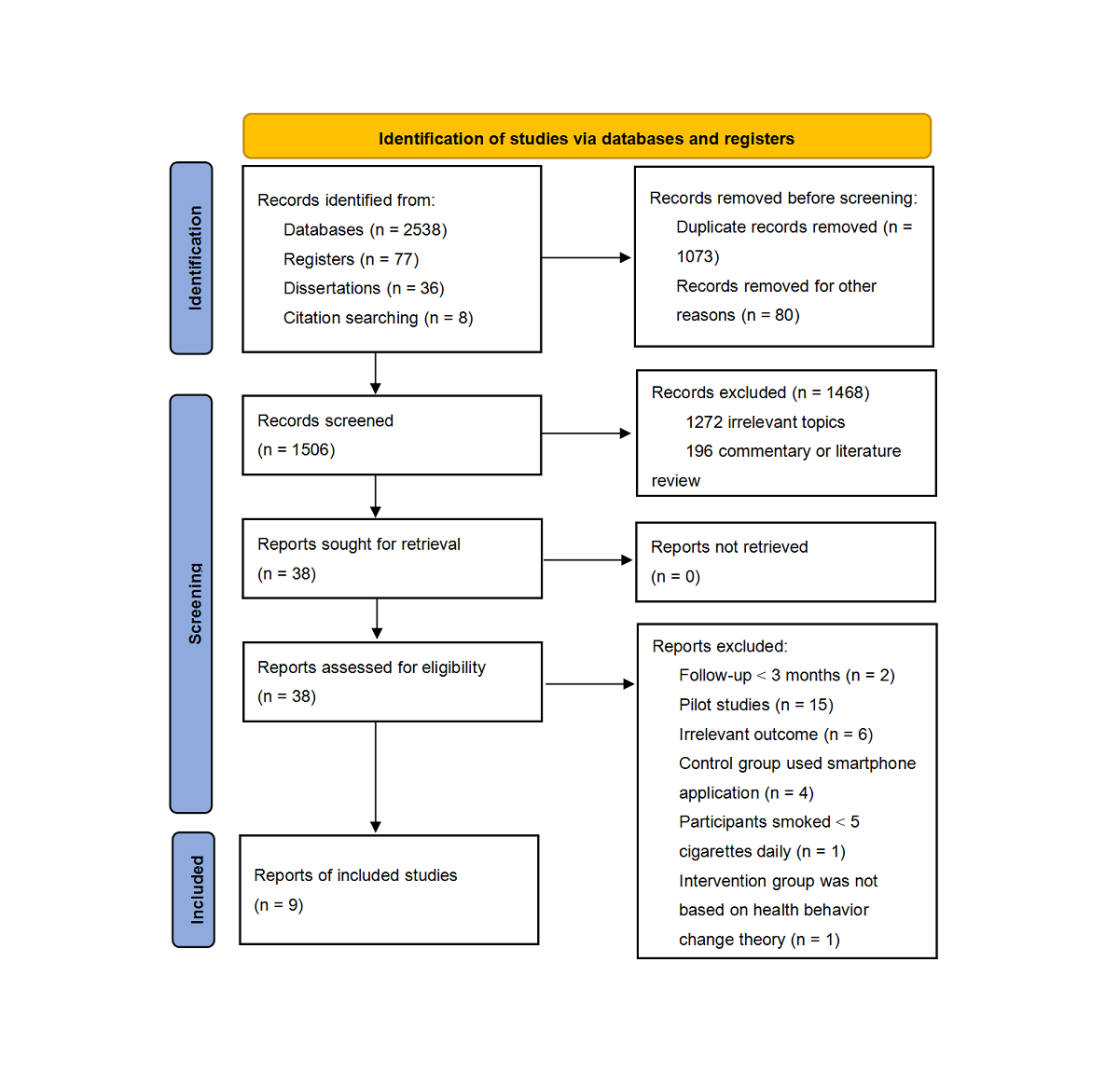
Flowchart of literature search. Databases referenced include Cochrane Library (n=521), Web of Science (n=997), PubMed (n=351), Embase (n=492), PsycINFO (n=131), China National Knowledge Infrastructure (n=19), and Wanfang database (n=27).

Our initial search yielded 2659 publications based on the defined search terms (Figure 1). After removing 1073 duplicates and irrelevant publications, a total of 1506 abstracts were screened, of which 1468 articles were excluded. The full-text reports for 38 potential studies were retrieved, of which 29 studies were excluded. According to the selection criteria, a final sample of 9 studies was included in this review. A flowchart of the study selection process is presented in Figure 1.

Nine RCTs were included in the meta-analysis, with a total of 12,967 participants. Sample sizes ranged from 240 to 5293 participants. The finally included trials were all published between 2018 and 2022, even though there was no year limit used as a search strategy. Among the included studies, 4 were conducted in European countries (the United Kingdom, Spain, France, or Switzerland) [21,22,24,26], 3 in the United States [20,23,25], 1 in Canada [19], and 1 in Japan [18]. Five studies reported biochemically verified measurements of smoking abstinence, and 4 studies relied on self-reported smoking abstinence. The mean age of the included participants ranged from 36 to 54 years old. The reported smoking abstinence rate among SASC groups varied from 7.8% to 63.9%. The comparators consisted of self-help booklets, web-based behavioral therapy, brief information or calculators (such as days without cigarettes or money saved), or scheduling interactions between patients and smoking cessation consultants. The details of compensation paid to the participants were reported in 5 studies [18,19,23,25,26], but not in the other 4 studies [20-22,24]. The characteristics of participants, intervention details, and outcome measures are presented in Tables 1 and 2.

**Table 1:** Characteristics of the included studies (N=9) by publication year.

Author (year)/country	Participants	Sample size (intervention group [IG]/control group [CG])	Age, mean (SD)	App name	Blinding	Theory	Control group intervention	Smartphone app–based smoking cessation plus pharmacotherapy
Baskerville 2018 [19]/Canada	Ages of 19 and 29 years	1599 (820/779)	N/A^a^ (aged 19 to 29 years)	Crush the Crave (CTC)	Double-blinded	Fiore’s practice guidelines; persuasive technology for behavior change	Self-Help Booklet “On the Road to Quitting”	No
Danaher 2019 [20]/United States	Adult smokers who wished to quit smoking, smoked ≥5 cigarettes per day for the previous 6-months	1271 (633/638)	44.9 (12.7)	MobileQuit 6-months duration	Nonindicated, web-based assessments. No difference in contact between arms	Cognitive behavior therapy; persuasive technology	Nonmobile device named QuitOnline (Designed for desktop, laptop, and tablet)	Part of participants
Affret 2020 [21]/France	Adult smokers who wish to quit smoking	2806 (1400/ 1406)	≤45 years 2163 (77.1); >45 years 643 (22.9%)	e-Tabac Info Service	Nonindicated	Relapse prevention model, behavioral change technique, motivation interview, social cognitive theory, the transtheoretical model	Current practices arm to visit a preexisting website page that listed smoking cessation resources	No
Carrasco-Hernandez 2020 [24]/Spain	Aged 18 years or older	240 (120/120)	IG: 48.38 (9.49) CG: 50.93 (10.85)	Social-Local-Mobile App	Single blinded - participants	Behavioral techniques	Pharmacological therapy with bupropion or varenicline plus behavioral therapy which was provided during face-to-face follow-up consultations	All patients
Garrison 2020 [23]/United States	Age 18-65 years	325 (143/182)	41 (12)	Craving to quit - 22 days	Single blinded-researcher	Mindfulness	Teach individuals to identify triggers and track the number of cigarettes they smoke	No
Masaki 2020 [18]/Japan	Nicotine-dependent adults	572 (285/287)	46 (11)	CureApp	Single blinded -participants	Evidenced behavior therapy	NRT^b^+Counseling+displaying the schedule of 5 visits during the 12-week treatment with a summary of objectives of each visit	All patients
Etter 2021 [22]/France and Switzerland	Age≥18 years daily smoker; Sets a target quit date within 1 month of enrollment, and commits to quit on this date	5293 (2639/2654)	Average age 36 years (range =19-79 years)	Stop-tabac	Double-blinded	Planned behavior, social cognitive theory and addiction theories. Guidelines	5 brief information pages and calculators	Part of participants
Houston 2022 [25]/United States	Smokers 18 years and older who are not preparing to quit, having at least 2 clinical visits in the past year	433 (213/220)	54 (13)	Take a Break	Double-blinded	Guideline, social cognitive theory and game mechanics concepts to engage participants in health behavior change	The comparison included Nicotine Replacement Therapy sampling only	Applied the NRT to all patients, but NRT is an optional choice for them
Webb 2022 [26]/United Kingdom	Smokers (aged ≥18), smoked >5 cigarettes per day for the past year	530 (265/265)	IG: 40 (12) CG: 42 (12)	Quit Genius	Single (Researchers were blinded to treatment allocation)	Cognitive behavioral therapy	Brief advice by trained research assistant: Ask, Advise, Act	All participants

^a^N/A: not applicable.

^b^NRT: nicotine replacement therapy.

**Table 2:** Characteristics of the included studies (N=9) by publication year.

Author (year)/country	Primary outcomes measurement	Secondary outcomes measurement	Compensation paid	Retention rate	Intervention engagement	Data collection method
Baskerville 2018 [19]/Canada	Continuous self-reported abstinence at 6 months	Self-reported nonsmoking in past 7 days; Self-reported nonsmoking in the past 30 days; satisfaction with the app	Participants were provided a Can $35^a^ incentive for registering to the study, and a raffle of an iPad Air tablet was used as an incentive to complete 6-months follow-up	IG: 354/820 (43.2%) CG: 371/779 (47.6%)	359/820 (43.8%) at 6- months (359 downloaded app)	Collected via a self-administered and web-based questionnaire
Danaher 2019 [20]/United States	Self-reported 7-day PPA^b^ at 3- and 6-months follow-up assessments	Engagement, helpfulness, satisfaction, usability	Not sure	6-months: IG: 359 (56.7%) CG: 329 (51.6%)	96% engage with the APP^c^. The overall number of app visits was 15.92 (15.79); the overall duration of app visits was 22.34 (30.46) minutes	A nationwide internet-based marketing campaign used Google AdWords, Reddit, Smokefree.gov, and ORI.org
Affret 2020 [21]/France	Self-reported 7-day PPA at 6 months	(1) continuous abstinence at 6-months; (2) continuous abstinence at 12-months; (3) minimum 24-hour point abstinence at 3-months; (4) minimum 30-day point abstinence at 12-months; (5) number and duration of quit attempts; (6) progress through the 4 modules in the intervention	Not sure	IG: 518/1400 (37.0%) CG: 602/1406 (42.8%)	3-months and 6-months usage rates for the app were 10.7% and 5.7%	When visiting their personal account on the French Mandatory National Health Insurance website, users were invited via a banner
Carrasco-Hernandez 2020 [24]/Spain	1-year smoking abstinence rate measured by exhaled carbon monoxide (CO) and cotinine urine test	(1) Quality of life; (2) International Physical Activity Questionnaire; (3) BMI	Not sure	IG: 51/120 (42.5%); CG: 45/120 (37.5%)	Low at 6-months follow up as paper’s figure indicated	Smokers were recruited during routine visits to our outpatient clinic
Garrison 2020 [23]/United States	7-day PPA from smoking at 6-months, verified by video-based CO-monitoring (<10 parts per million [ppm])	Smoking cigarettes per day, craving, and mindfulness, from baseline and 6-months surveys	Paying $10 for the end of treatment survey, $20 each for surveys at 3- and 6-months follow-up, and $0.50 per experience sampling “check-in”	Retention was defined as answering the primary outcome questions at 6-months-72.60%	As completed 60% of MMT-ES modules or checking in on 60% of treatment days, 53.1% completed week 1, 41.3% in week 2, and 28.7% in week 3	Recruitment sources were as follows: Google advertisements, word of mouth or other, Facebook posts, Twitter, et al All surveys were automated
Masaki 2020 [18]/Japan	Validated CAR^d^ from 9-weeks to 24-weeks measured by CO Tester	(1) Biochemically validated CAR from weeks 9 to 12 and weeks 9 to 52; (2) 7-day PPA at weeks 4, 8, 12, 24, and 52; (3) withdrawal symptoms; (4) cravings; (5) misperceptions about smoking; (6) Time to first lapse after the quit date; (7) The usage rate of either the intervention or the control app; (8) The presence of product problems or adverse events	Compensation of US $90 per visit may have impacted both app engagement and quitting success, although compensation was provided to both groups	IG: 89.1% (254/285) CG: 87.8% (252/287)	99.6% (252/253) at week 24	Recruited nicotine-dependent adults who visited outpatient clinics to receive smoking cessation treatment
Etter 2021 [22]/France and Switzerland	Self-reported smoking cessation after 6-months (no puff of tobacco in the past 4 weeks)	Self-reported use of nicotine medications	Not sure	IG: 662/2639 (25.1%); CG: 745/2654 (28.1%)	15% of participants used it during the whole 6 months	Once on the app stores, participants downloaded the app
Houston 2022 [25]/United States	Time to first quit attempt (duration from Take a Break experience to primary outcome) and CO level–verified smoking cessation at 6-month follow-up	Smoking Self-Efficacy Questionnaire; 6-months prevalent abstinence was self-reported and verified by a CO meter	All compensation received within 48 hours upon completion of the particular task (ie, after in-person session, after telephone survey). Compensation was in the form of Amazon gift cards, in $25 and $50 denominations. Study participants at Reliant Medical Group will receive checks (mail to them after each visit) in the same amounts as Amazon gift cards	IG: 160/213 (75.1%); CG: 171/220 (77.7%)	112 (53%) completed daily quizzes in the first week and 73 (34%) completed quizzes in the following second week. 159 (75%) using the coping mini-games	Participants were recruited from individuals who had at least 1 visit in the preceding 12-months to participating clinical sites
Webb 2022 [26]/United Kingdom	Abstinence was verified using a random sample of participants with CO breath testing of <5 parts per million (n = 280)	7-day PPA at 26-week and 52-week follow-up, consecutive 7-day PPA at 26-weeks and 52-weeks follow-up time points. Sustained abstinence; the number of quit attempts at 26-weeks and 52-weeks post-Quit Date	Participants received £10^e^ to offset travel costs and were compensated for each follow-up data collection visit as follows: £10^e^ (26 weeks) and £20^e^ (4- and 52-week follow-ups)	Week 52: IG 209 (79%); CG 212 (80%)	42.8% of the Quit Genius participants completed the CBT essentials	Participants were recruited via social media and referrals from primary care practices in the United Kingdom between January and November.

^a^CAD $1=USD $0.94 on July 1, 2019.

^b^PPA: point prevalence abstinence.

^c^Among the MobileQuit participants, 90.0% (570/633) accessed the app multiple times, 6.0% (38/633) accessed once, and 4.0% (25/633) never accessed.

^d^CAR: continuous abstinence rate.

^e^GBP £1=USD $1.32 US Dollar on December 31, 2019.

### Risk of Bias

Bias was assessed based on the Cochrane risk of the bias assessment tool, RoB 2 [31,32]. Three studies were double-blinded [19,22,25], 4 studies used the single-blinded approach of either the participants [18,24] or study staff [23,26], and 2 studies did not report the blinding method [20,21]. In fact, given the nature of the SASC interventions, researchers could not prevent the participants from knowing their group assignment.

**Figure 2:**
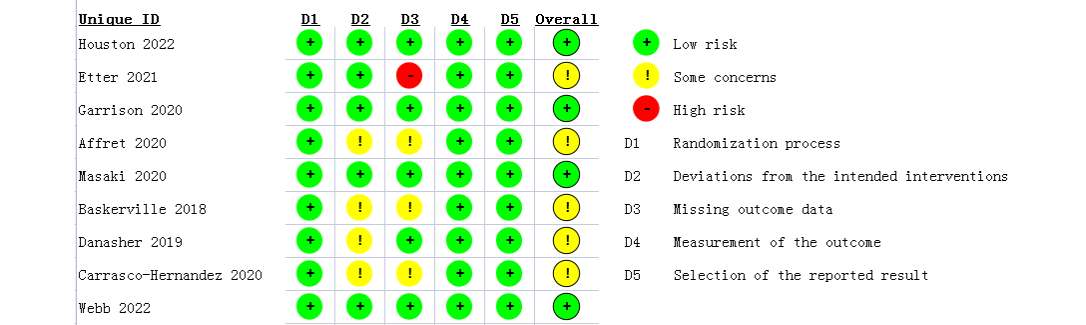
Analysis of the risk of bias in each included trial.

Several sources of bias were found. The rate of loss to follow-up was high, and the representativeness of the research results was biased. Four studies reported that the retention rate was less than 50%, reflecting moderate risk. More details are shown in Figure 2. Even though 4 included studies used self-report to measure smoking abstinence, we did not consider it a high source of bias in the measurement of the outcome since biochemical validation is not always necessary in smoking cessation studies because levels of misrepresentation are generally low (0%-8.8%) [30].

**Figure 3:**
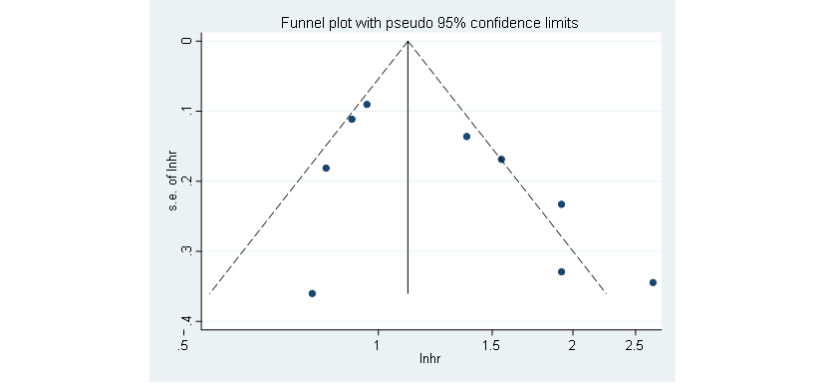
Funnel plot displaying the probable publication bias.

As shown in Figure 3, the funnel plot was conducted to identify whether publication bias existed. The funnel plot was generally symmetrical (Egger test, *P*=.10; Begg test, *P*=.12), which implied no publication bias existed in the included studies.

### Results of the Meta-analysis

**Figure 4:**
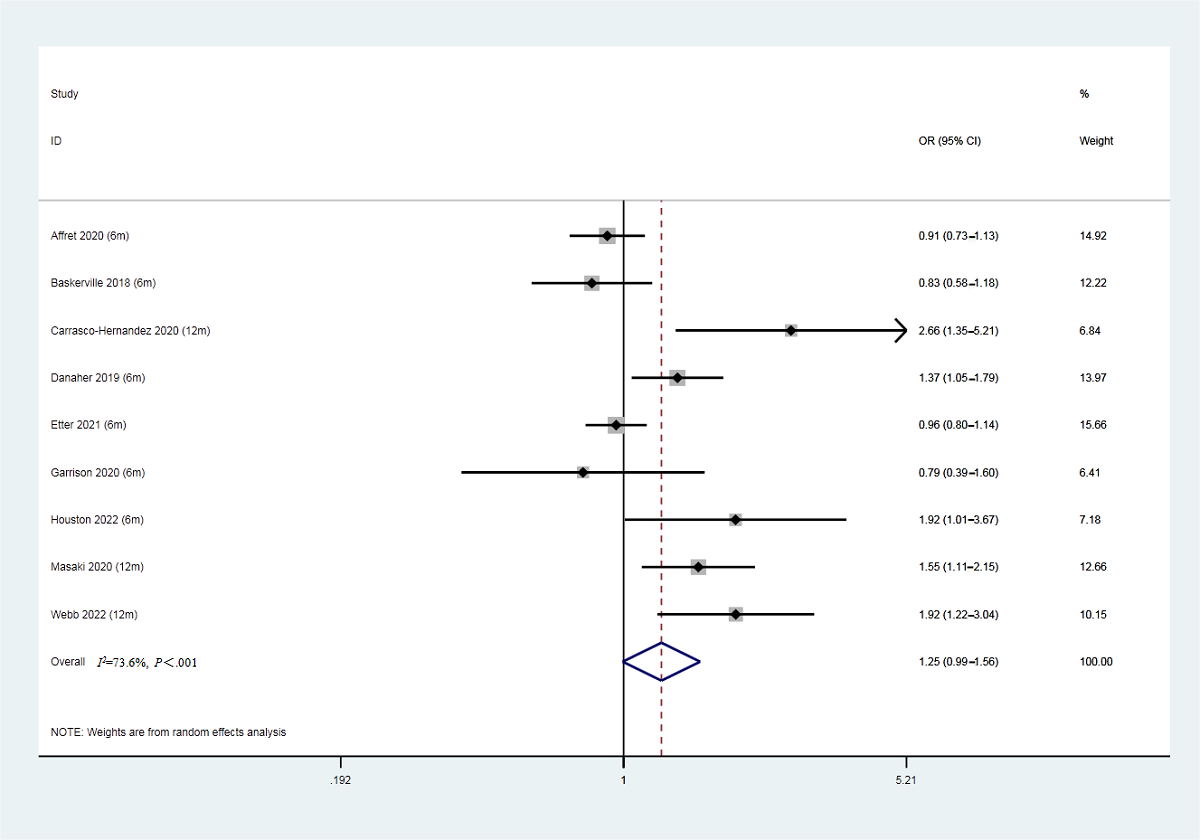
Forest plot of smoking abstinence rates (9 studies, N=12,967). OR: odds ratio.

The smoking abstinence rate among the 9 enrolled studies is displayed in Figure 4. There was no significant difference in the smoking abstinence rate between the experimental and control groups (OR 1.25, 95% CI 0.99-1.56, *P*=.06, *I*^2^=73.6%). A leave-one-out sensitivity analysis was performed to examine the results. As displayed in Table 3, the sensitivity analysis shows that our results did not change in the heterogeneity outcomes, with the *I*^2^ ranging from 66% to 76%.

**Table 3:** Leave-one-out sensitivity analysis of the smoking abstinence with intervention and control groups by publication year.

Study if excluded	OR^a^	95% CI	*P* value	*I* ^2^
Baskerville [19] (6 months)	1.32	1.03-1.69	.03	75%
Danaher [20] (6 months)	1.23	0.96-1.59	.11	75%
Affret [21] (6 months)	1.32	1.02-1.72	.03	73%
Carrasco-Hernandez [24] (12 months)	1.17	0.96-1.42	.12	66%
Garrison [23] (6 months)	1.29	1.02-1.63	.04	76%
Masaki [18] (12 months)	1.21	0.95-1.54	.11	72%
Etter [22] (6 months)	1.32	1.01-1.73	.04	73%
Houston [25] (6 months)	1.20	0.96-1.52	.11	75%
Webb [26] (12 months)	1.18	0.94-1.48	.15	71%

^a^OR: odds ratio.

### Subgroup Analysis of Smartphone App–Based Intervention at Different Follow-Up Time Points

**Figure 5:**
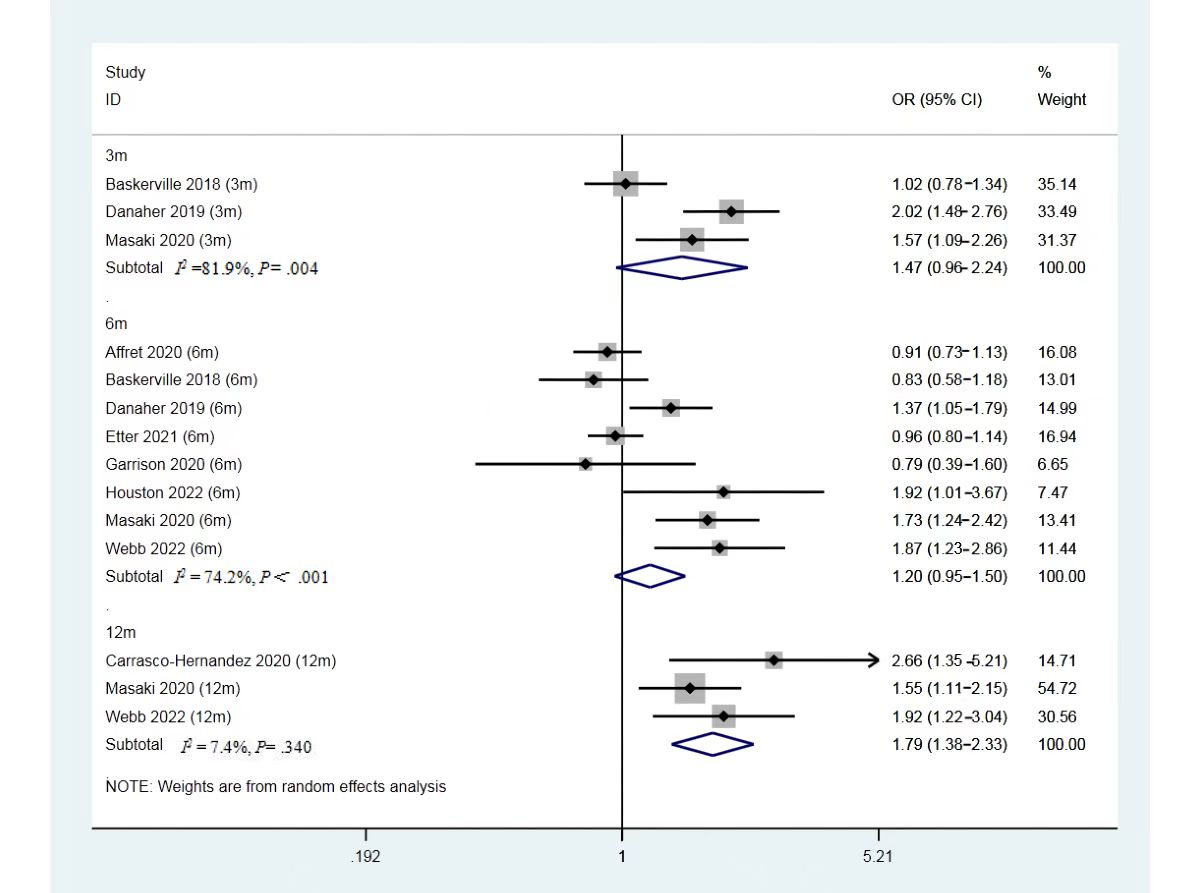
Forest plot for the rate of smoking abstinence at 3-, 6-, and 12-month follow-up. OR: odds ratio.

Referring to Figure 5, subgroup analysis was performed to further explore the differences in effectiveness by the duration of follow-up. The 4 studies that reported 3-month results (3 studies, N=3442) found no significant differences between the SASC group and the control group (OR 1.47, 95% CI 0.96-2.24, *P*=.08, *I*^2^=81.9%). Moreover, at the 6-month follow-up (8 studies, N=12,727), no statistically significant differences were observed between groups (OR 1.20, 95% CI 0.95-1.50, *P*=.12, *I*^2^=74.2%). Studies that reported the smoking abstinence rate at 12 months (3 studies, N=1342) found statistically significant differences between groups (OR 1.79, 95% CI 1.38-2.33, *P*<.001, *I*^2^=7%) [18,24,26]. However, among all 3 studies, both the intervention and control groups received pharmacotherapy. On top of that, the intervention groups also received smartphone app–based interventions, while the comparator group only received pharmacotherapy. In this way, the effect of smartphone app–based intervention was observed.

### Subgroup Analysis of Solely SASC Interventions or SASC Combined With Pharmacotherapies

**Figure 6:**
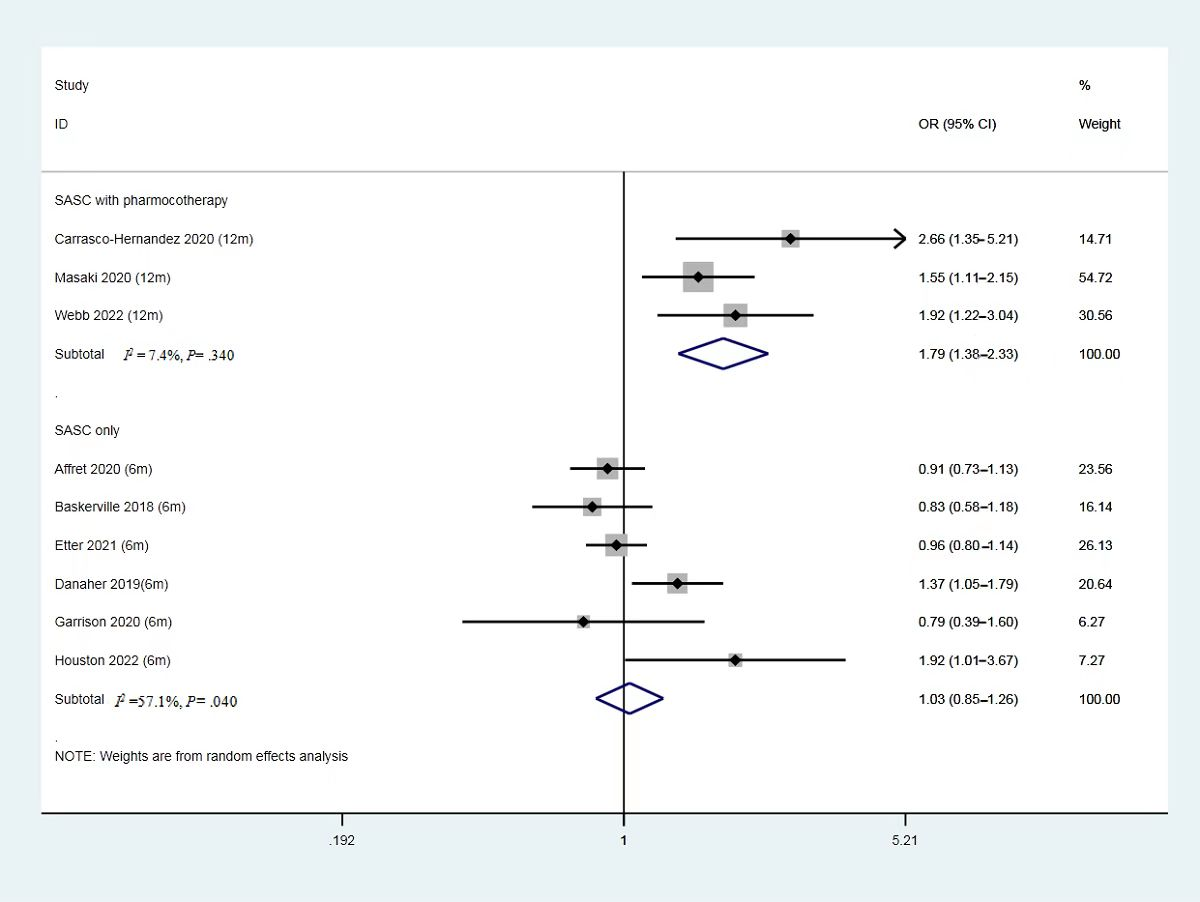
Forest plot of smoking abstinence rate of solely SASC intervention and SASC-assisted pharmacotherapy intervention. SASC: smartphone app–based smoking cessation; OR: odds ratio.

Referring back to the elements of the interventions (Multimedia Appendix 2), it shows 6 studies delivered the health behavior change theories driving smoking cessation intervention by smartphone apps alone; however, 3 studies used the apps to augment pharmacological cessation therapies. As shown in Figure 6, 6 studies with smartphone app–based interventions alone reported no significant differences in effectiveness between the 2 groups (OR 1.03, 95% CI 0.85-1.26, *P*=.74, *I*^2^=57.1%). However, intervention groups combining smartphone-based app use and pharmacologic therapies for smoking cessation (3 studies, N=1342) reduced the chance of quitting smoking. Researchers conducted the interventions via smartphone apps, coupled with pharmacotherapies and consultations for the participants. On the other hand, comparators were pharmacotherapies and consultations for the participants. The smartphone app combined with pharmacotherapies and consultation groups increased the smoking abstinence rate compared with the comparators (OR 1.79, 95% CI 1.38-2.33, *P*<.001, *I*^2^=7%).

### Subgroup Analysis of Different Levels of Adherence

**Figure 7:**
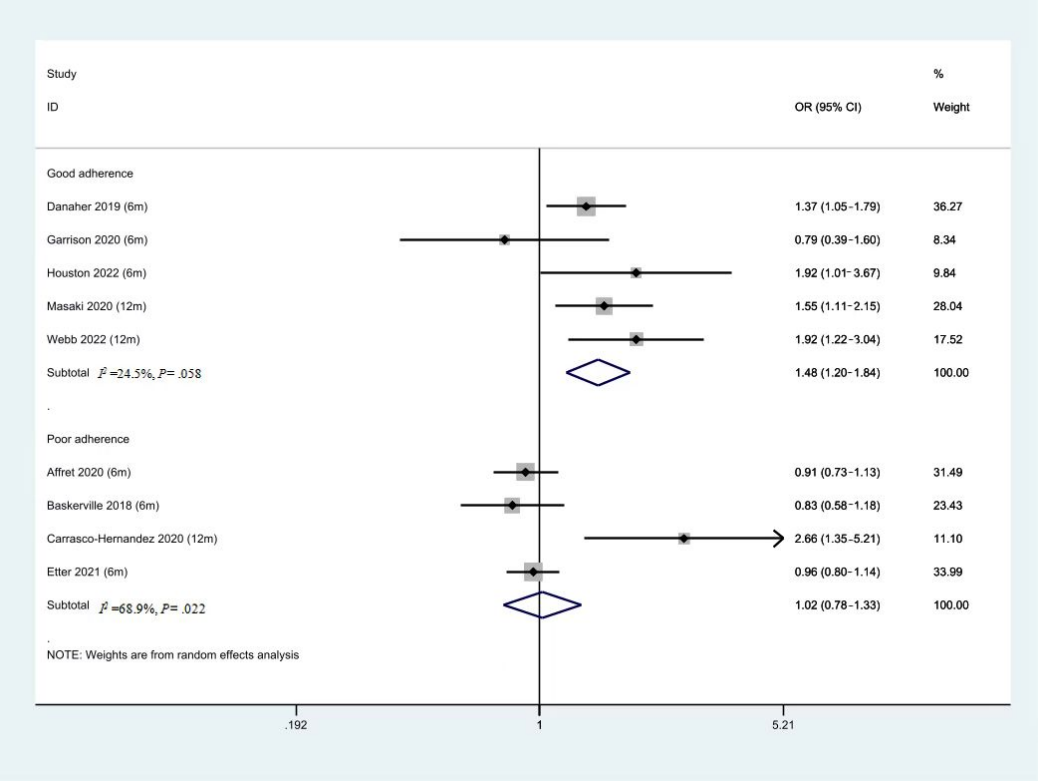
Forest plot of smoking abstinence rate of intervention with good or poor adherence. OR: odds ratio.

As shown in Figure 7 (5 studies, N=3029), 5 studies with higher adherence rates (more than 50%) showed better effectiveness of SASC (OR 1.48, 95% CI 1.20-1.84, *P*<.001, *I*^2^=24.5%). The other 4 studies (N=9938) with poor adherence rates (less than 50%) showed limited effectiveness of SASC (OR 1.02, 95% CI 0.78-1.33, *P*=.88, *I*^2^=68.9%).

### Subgroup Analysis of Different Recruitment Strategies

**Figure 8:**
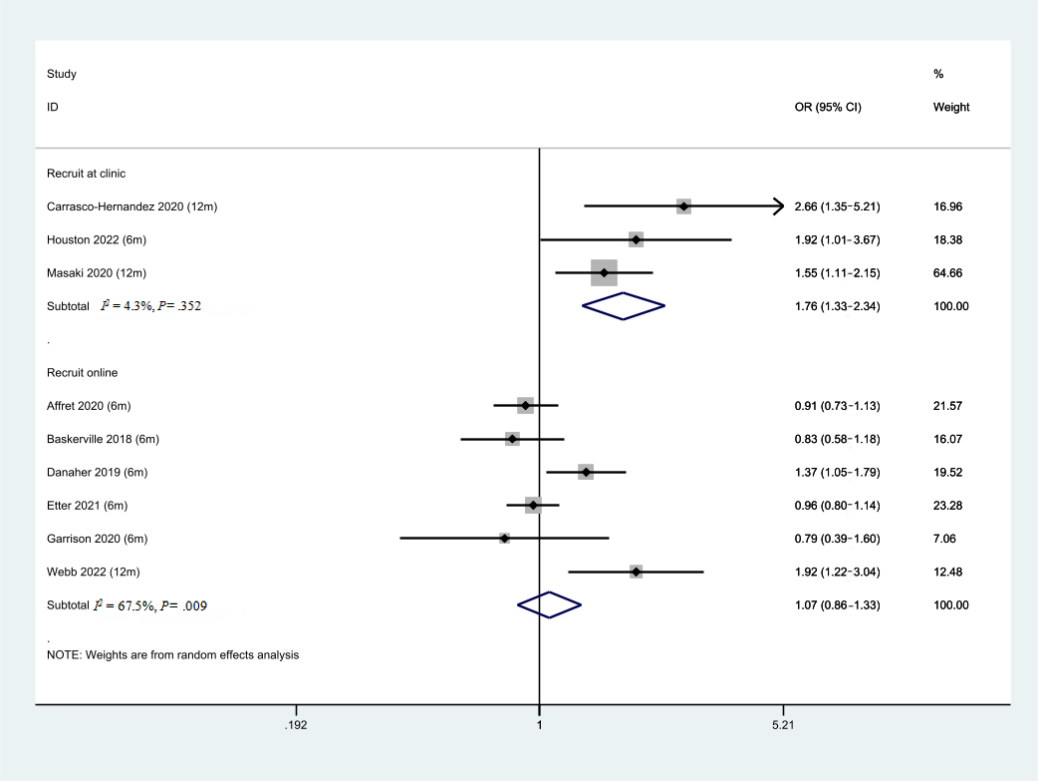
Forest plot of smoking abstinence rate for different recruitment strategies. OR: odds ratio.

As shown in Figure 8, the 3 studies where participant recruitment occurred in clinics showed better effectiveness of SASC (OR 1.76, 95% CI 1.33-2.34, *P*<.001, *I*^2^=4.3%) compared to the 6 studies that recruited participants through the web (OR 1.07, 95% CI 0.86-1.33, *P*=.55, *I*^2^=67.5%).

### Subgroup Analysis of Different Outcome Measure Methods

**Figure 9:**
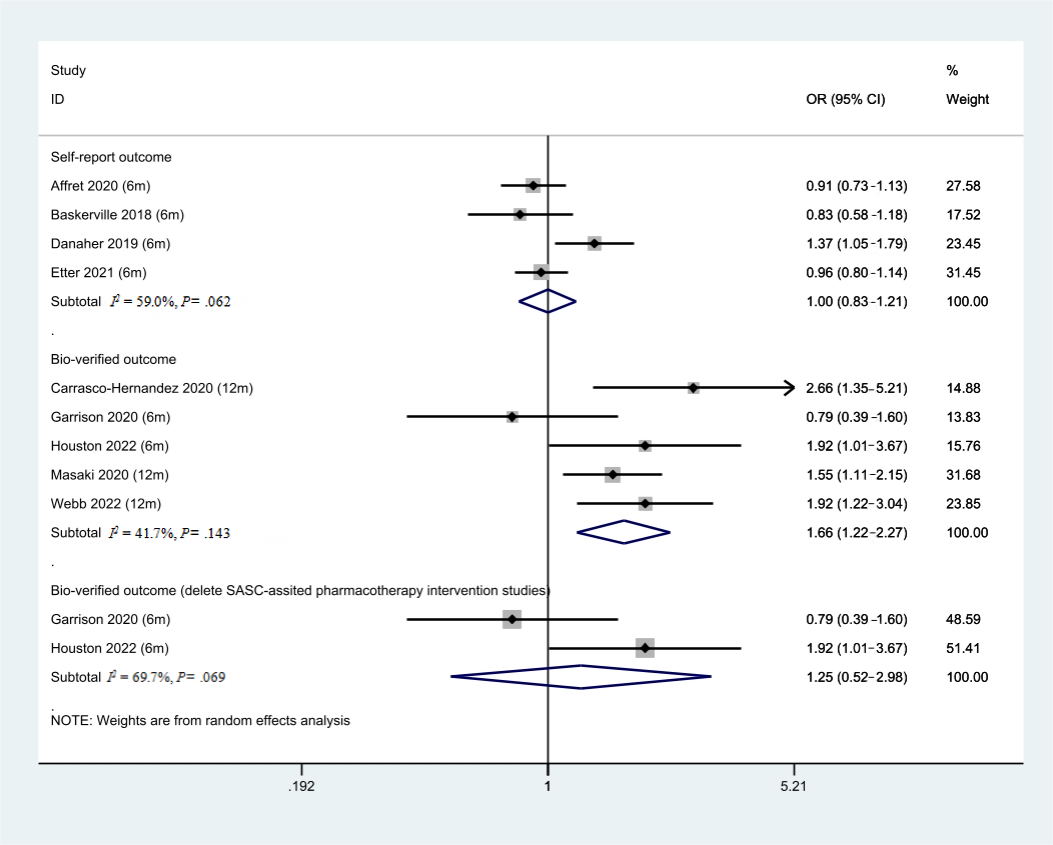
Forest plot of different outcome measurements. OR: odds ratio.

As shown in Figure 9, five studies used self-report as the measure of smoking abstinence outcome. The pool effects of these studies showed limited effectiveness of SASC intervention (OR 1.00, 95% CI 0.85-1.18, *P*=.99, *I^2^*=59%). However, 5 studies used the bio-verified method (exhaled carbon monoxide or cotinine urine test) to measure smoking abstinence. The pool effects of the 5 studies showed the effectiveness of SASC (OR 1.46, 95% CI 1.11-1.92, *P*=.001, *I^2^*=53%). All 3 SASC-assisted pharmacotherapy intervention studies used the bio-verified method to measure smoking abstinence. To minimize the intervention factors, we explored the pooled effect of bio-verified outcomes studies by removing those 3 studies. There were no significant differences in effectiveness between the 2 groups (OR 1.21, 95% CI 0.57-2.59, *P*=.62, *I*^2^=69%).

## Discussion

### Principal Findings

We conducted a systematic review and meta-analysis of 9 relatively high-quality RCTs through a comprehensive database search to determine the impact of SASC interventions. A total of 12,967 adult participants were included in this study. Although our findings failed to prove that SASC interventions alone were effective for smoking abstinence, improved smoking abstinence was achieved when SASC interventions were used to augment smoking cessation pharmacotherapy. In addition, improved smoking abstinence was shown for studies with participants recruited from the clinic rather than through the web. The study also found that the method for measuring the outcome of smoking cessation (self-report or verified biological measures) did not necessarily impact outcome assessment, which is consistent with previous reports [30]. Compared to previous meta-analyses of SASC interventions, this study identified more reports of RCTs with larger sample sizes, allowing us to draw more accurate and valid inferences. In addition, bias was assessed in 5 distinct domains via RoB 2. Within each domain, we answered the signaling questions. These answers lead to judgments of “low risk of bias,” “some concerns,” or “high risk of bias.” Major biases in the included studies were the lack of blinding; given the nature of the intervention and the control, some researchers could not blind participants to treatments. Thus, the results may reflect participant expectation bias. Furthermore, not all research programs could capture consistent participant-level data on intervention adherence, which may increase the nonrespondent bias.

Based on our meta-analysis, there are several possible reasons why SASC interventions alone showed lower smoking abstinence rates. First, adherence to smartphone app–based interventions may be the main reason for the failure of smoking cessation. As shown in our results, lower abstinence rates resulted from poor adherence to the intervention. On the contrary, Masaki and Webb’s studies [18,26] reported that SASC-assisted pharmacotherapy groups had higher smoking abstinence rates, possibly because SASC intervention could increase the participants’ understanding of behavioral intervention methods and provide smoking cessation information at any time. In addition, as shown in our results, improved smoking abstinence was achieved if the study participants were recruited from clinics rather than through the web. Smokers who took smoking cessation medications had face-to-face communication and clinical follow-up with clinic smoking cessation consultants, which in turn may have increased smokers’ motivation and frequency of using smoking cessation apps.

Poor adherence may result from the ubiquitous low engagement of the smartphone app–based intervention, which is not uncommon and consistent with previous health behavior change studies [34-36]. Smartphone-based interventions may have little effect if individuals do not adhere to the intervention protocol [29]. We did not do the meta-analysis based on engagement because the measures of engagement with the intervention were inconsistent and inadequate [37,38], as they did not monitor the number of log-ins or time spent using each module to verify the engagement. Although there is no consensus on the benchmarks for reporting eHealth engagement, engagement could be increased through various mechanisms that depend heavily on the characteristics of current technology, users’ characteristics (such as literacy and habits), and their environmental factors. Therefore, to raise adherence to SASC interventions, it is highly suggested to consider improving the app to support smokers’ engagement and setting benchmarks for reporting smartphone-based interventions. In addition, the financial compensation paid may also increase the motivation to complete the intervention.

Secondly, ineffective intervention may result from not fully exploiting features. The features of smartphone apps that have the potential to support behavior change and customization, such as real-time feedback, individualized messaging, or interaction, may have increased the effectiveness and individualization of app-based interventions. Etter’s study [22] provided automated, individually tailored counseling messages based on the user’s profile, regularly sent for 6 months, immediate feedback during episodes of craving and tobacco withdrawal symptoms, and a module on nicotine therapy that includes personalized feedback and follow-up. Webb’s study [26] provided personalized CBT-based support via the in-app chat and phone, with a 30-min phone call at baseline, discussing their individualized quit plan and methods of using NRT. Affret et al’s [21] study provided personalized messages relating to the answers to the different questionnaires. The details of the customized features were various. There was a lack of representativeness and a precise definition of the individualized in the real sense. We did not check the effectiveness based on the extent of the individualization feature. In fact, with the current level of artificial intelligence technology development, computer engineers have been able to provide instantaneous ecological momentary assessment methods, predict smoking risks based on user data, and push timely information based on algorithms. However, researchers may face numerous barriers when offering SASC intervention; for instance, research participants may worry about their privacy or consider cumbersome data entry a burden. To date, only a few SASC intervention studies have achieved individualization and timely information response.

Although smartphone app–based intervention alone did not achieve superior efficacy, higher effect sizes were found in studies that combined smartphone app–based intervention with pharmacotherapy. There might be 2 reasons for this, which include the fact that SASC intervention is not limited by time or region, as app users can receive the intervention according to their schedule. Second, the smokers who received pharmacotherapy are regularly followed up by physicians, which may increase the stickiness of app use. In addition, we found that the SASC is more effective at 12 months than shorter follow-ups. This could be because all 12-month follow-up studies are the same studies that intervene with the participants with the SASC combined with pharmacotherapy. To assist smokers in quitting smoking in the future, it is recommended to use SASC intervention in smokers who receive smoking cessation drug treatment. Future studies can reflect on how to balance the frequency of the combination of outpatient follow-up and SASC to maximize the smoking cessation rate with the minimized cost of human medical resources.

### Strengths and Limitations

The strengths of our meta-analysis are that all of the included studies had large sample sizes. Although smoking cessation research based on smartphone apps is still emerging, our research team included many high-quality RCTs, which allowed us to draw more accurate and valid inferences. Moreover, all the included studies conducted behavioral theory-driven interventions, which could enhance the delivery of the intervention and boost its effects on participants. However, our study also has some limitations. First, heterogeneity among the included studies still exists even after we applied the subgroup and sensitivity analyses. Second, subgroup analysis based on different follow-up times had been conducted, but the number of included studies was even lower. Third, although all smoking cessation interventions were applied by smartphone apps, the specific interventions may vary widely between the studies. Fourth, low adherence to the SASC intervention brought increased threats to the research’s reliability and validity. Lastly, the abstinence outcome criteria used in the included studies varied, such as continuous smoking abstinence and 7-day point prevalence smoking abstinence indicators, which may also increase the heterogeneity of the pool studies. Future work on improving adherence to SASC intervention is warranted to evaluate the future effect of SASC intervention. It is recommended to use SASC intervention in the future for smokers who receive smoking cessation drug treatment to assist smokers to quit smoking and to continue to refine the frequency of the combination of outpatient follow-up and SASC to maximize the smoking cessation rate and optimize the cost of human medical resources. Finally, although the promise of the engagement report of the SASC intervention has not yet been fully realized, future research may provide insight into how to target the engagement of smartphone app–based interventions, which may affect intervention adherence and smoking cessation abstinence.

### Conclusions

In conclusion, our study is confined to determining the effectiveness of smartphone-based apps in smoking cessation intervention within the boundaries of existing evidence. The current meta-analysis did not reveal superior effects for stand-alone smartphone-based apps. However, smartphone app–based interventions may be the most effective tool affiliated with pharmacotherapeutics. Furthermore, smartphone app–based interventions with good adherence showed improved smoking abstinence. More rigorously designed RCTs with robust engagement reporting should be encouraged to test the effectiveness of smartphone-based app interventions for smoking abstinence.

## Appendix

Multimedia Appendix 1 Search strategies of PubMed, Embase, and the Cochrane Library.

Multimedia Appendix 2 PRISMA (Preferred Reporting Items for Systematic Reviews and Meta-Analyses) checklist.

## Abbreviations

OR: odds ratio
RCT: randomized controlled trial
SASC: smartphone app–based smoking cessation
