# Supplementary material for: The Effectiveness of Smartphone App–Based Interventions for Assisting Smoking Cessation: Systematic Review and Meta-analysis
Source: J Med Internet Res. 2023 Apr 20;25:e43242. doi: 10.2196/43242 (PMC10160935; doi:10.2196/43242)
Supplement: Multimedia Appendix 1 [file jmir_v25i1e43242_app1.docx]

**Searching strategies in Pubmed database**

| Steps | Search Terms | Results |
| --- | --- | --- |
| #1 | ((((((((((((((((((telemedicine[MeSH Terms]) OR (ehealth[Title/Abstract])) OR (mhealth[Title/Abstract])) OR (digital health[Title/Abstract])) OR (mobile health[Title/Abstract])) OR (telehealth[Title/Abstract])) OR (mobile phone [Title/Abstract])) OR (mobile device [Title/Abstract])) OR (cellphone [Title/Abstract])) OR (smartphone [Title/Abstract])) OR (smartphone apps[Title/Abstract])) OR (cellphone apps[Title/Abstract])) OR (mobile apps[Title/Abstract])) OR (smartphone based[Title/Abstract])) OR (cellphone based[Title/Abstract])) OR (mobile phone based[Title/Abstract])) OR (mobile applications[MeSH Terms])) OR (portable electronic app*[Title/Abstract])) OR (portable software app*[Title/Abstract]) | 78,829 |
| #2 | (((((((smoking cessation[MeSH Terms]) OR (smoking abstinence[Title/Abstract])) OR ('tobacco abstinence[Title/Abstract])) OR (tobacco use cessation[Title/Abstract])) OR (tobacco quitting[Title/Abstract])) OR (quit* smok*[Title/Abstract])) OR (give up smok*[Title/Abstract])) OR (stop* smok*[Title/Abstract]) | 35,683 |
| #3 | (randomized controlled trial [pt] OR controlled clinical trial [pt] OR randomized [tiab] OR  placebo [tiab] OR drug therapy [sh] OR randomly [tiab] OR trial [tiab] OR groups [tiab]) NOT (animals [mh] NOT humans [mh]) | 4,644,805 |
| #4 | #1 AND #2 AND #3 | 351 |

**Searching strategies in Embase database**

| Steps | Search Terms | Results |
| --- | --- | --- |
| #1 | 'telemedicine'/exp OR ehealth:ab,ti OR mhealth:ab,ti OR 'digital health':ab,ti OR 'mobile health':ab,ti OR telehealth:ab,ti OR 'mobile phone$':ab,ti OR 'mobile device$':ab,ti OR cellphone$:ab,ti OR smartphone$:ab,ti OR 'smartphone app$':ab,ti OR 'cellphone app$':ab,ti OR 'mobile app$':ab,ti OR 'smartphone based':ab,ti OR 'cellphone based':ab,ti OR 'mobile phone based':ab,ti OR 'mobile applications'/exp OR 'portable electronic app$':ab,ti OR 'portable software app$':ab,ti | 124,806 |
| #2 | 'smoking cessation'/exp OR 'smoking abstinence':ab,ti OR 'tobacco abstinence':ab,ti OR 'tobacco use cessation':ab,ti OR 'tobacco quitting':ab,ti OR 'quit$ smok$':ab,ti OR 'giv$ up smok$':ab,ti OR 'stop$ smok$':ab,ti | 64,381 |
| #3 | 'clinical trial'/de OR 'randomized controlled trial'/de OR 'randomization'/de OR 'single blind procedure'/de OR 'double blind procedure'/de OR 'crossover procedure'/de OR 'placebo'/de OR 'prospective study'/de OR 'randomi?ed controlled' NEXT/1 trial* OR rct OR 'randomly allocated' OR 'allocated randomly' OR 'random allocation' OR allocated NEAR/2 random OR single NEXT/1 blind* OR double NEXT/1 blind* OR (treble OR triple) NEAR/1 blind* OR placebo* | 2,633,202 |
| #4 | #1 AND #2 AND #3 | 492 |

**Searching strategies in Cochrane database**

ID Search Hits

#1 MeSH descriptor: [Telemedicine] explode all trees 2862

#2 (ehealth):ti,ab,kw OR (mhealth):ti,ab,kw OR (digital health):ti,ab,kw OR (mobile health):ti,ab,kw OR (telehealth):ti,ab,kw 11004

#3 (mobile phone*):ti,ab,kw OR (mobile device*):ti,ab,kw OR (cellphone*):ti,ab,kw OR (smartphone*):ti,ab,kw OR (smartphone*):ti,ab,kw 8631

#4 (smartphone app*):ti,ab,kw OR (cellphone app*):ti,ab,kw OR (smartphone based):ti,ab,kw OR (cellphone based):ti,ab,kw OR (mobile phone based):ti,ab,kw 5259

#5 (portable electronic app*):ti,ab,kw OR (portable software app*):ti,ab,kw 211

#6 MeSH descriptor: [Mobile Applications] explode all trees 839

#7 #1 OR #2 OR #3 OR #4 OR #5 OR #6 17758

#8 MeSH descriptor: [Smoking Cessation] explode all trees 4194

#9 (smoking abstinence):ti,ab,kw OR (tobacco abstinence):ti,ab,kw OR (tobacco use cessation):ti,ab,kw OR (tobacco quitting):ti,ab,kw OR (quit* smok*):ti,ab,kw 8734

#10 (giv* up smok*):ti,ab,kw OR (stop* smok*):ti,ab,kw 3492

#11 #8 OR #9 OR #10 11408

#12 #7 AND #11 638

#13 (Randomized controlled trial) OR ("randomized-controlled trials") OR (Random allocation) OR (Double blind method) OR (Single blind method) 1102850

#14 #12 AND #13 521

**Searching strategies in Psychoinfo database**

| Steps | Search Terms | Results |
| --- | --- | --- |
| #1 | MA telemedicine OR TX ( ehealth or telehealth ) OR TX mhealth OR TX digital health OR TX mobile health OR MA mobile applications OR TX mobile phone* OR TX mobile device* OR TX cellphone* OR TX smartphone* OR TX ( 'smartphone based' OR ‘cellphone based’ OR 'mobile phone based' OR 'portable electronic app*' OR 'portable sofware app*' ) OR TX cellphone based | 21,226 |
| #2 | MA smoking cessation OR TX smoking abstinence OR TX tobacco abstinence OR TX tobacco use cessation OR TX tobacco quitting OR TX quit* smok* OR TX giv* up smok* OR TX stop* smok* | 15,412 |
| #3 | SU.EXACT("Treatment Effectiveness Evaluation") OR SU.EXACT.EXPLODE("Treatment Outcomes") OR SU.EXACT("Placebo") OR SU.EXACT("Followup Studies") OR placebo* OR random* OR "comparative stud*" OR clinical NEAR/3 trial* OR research NEAR/3 design OR evaluat* NEAR/3 stud* OR prospectiv* NEAR/3 stud* OR (singl* OR doubl* OR trebl* OR tripl*) NEAR/3 (blind* OR mask*) | 262,548 |
| #4 | #1 AND #2 AND #3 | 131 |

**Searching strategies in ISI Web of Science**

| Steps | Search Terms | Results |
| --- | --- | --- |
| #1 | (((((((((((TS=(TELEMEDICINE)) OR TS=( mhealth)) OR TS=(digital health)) OR TS=(mobile health)) OR TS=( telehealth)) OR TS=(mobile phone*)) OR TS=(mobile device*)) OR TS=(cellphone*)) OR TS=(smartphone*)) OR TS=(smartphone app*)) OR TS=(cellphone app*)) OR TS=(mobile app*) | 383,703 |
| #2 | (((((TS=(smartphone based)) OR TS=(cellphone based)) OR TS=(mobile phone based)) OR TS=(mobile applications)) OR TS=(portable electronic app*)) OR TS=(portable software app*) | 127,413 |
| #3 | #1 OR #2 | 390,647 |
| #4 | (((((((TS=(smoking cessation)) OR TS=(smoking abstinence)) OR TS=('tobacco abstinence)) OR TS=(tobacco use cessation)) OR TS=(tobacco quitting)) OR TS=(quit* smok*)) OR TS=(giv* up smok*)) OR TS=(stop* smok*) | 87,994 |
| #5 | TS= clinical trial* OR TS=research design OR TS=comparative stud* OR TS=evaluation stud* OR TS=controlled trial* OR TS=follow-up stud* OR TS=prospective stud* OR TS=random* OR TS=placebo* OR TS=(single blind*) OR TS=(double blind*) | 10,510,391 |
| #6 | #3 AND #4 AND #5 | 997 |
